# Supplementary material for: Comparative genomics provides new insights into the diversity, physiology, and sexuality of the only industrially exploited tremellomycete: Phaffia rhodozyma
Source: BMC Genomics. 2016 Nov 9;17:901. doi: 10.1186/s12864-016-3244-7 (PMC5103461; doi:10.1186/s12864-016-3244-7)
Supplement: Additional file 6: — List of orphan genes with links to PFAM (related to Additional file 1: Table S1). (ZIP 1428 kb) [file 12864_2016_3244_MOESM6_ESM.zip › PFAM_UNIQUE/G01488_P.html]

#                                                                            --- full sequence --- -------------- this domain -------------   hmm coord   ali coord   env coord
  
# target name        accession   tlen query name           accession   qlen   E-value  score  bias   #  of  c-Evalue  i-Evalue  score  bias  from    to  from    to  from    to  acc description of target
  
#------------------- ---------- ----- -------------------- ---------- ----- --------- ------ ----- --- --- --------- --------- ------ ----- ----- ----- ----- ----- ----- ----- ---- ---------------------
  
G01488\_P             -            516 BUD22                PF09073.5    432      0.02   12.6   3.0   1   1   0.00023     0.031   12.0   3.0   138   205   382   449   370   488 0.75 - BUD22  
G01488\_P             -            516 Daxx                 PF03344.10   713      0.33    8.0   8.6   1   1    0.0055      0.45    7.6   8.6   403   503   357   450   352   497 0.38 - Daxx Family  
G01488\_P             -            516 DUF1777              PF08648.7    180       2.4    6.5  10.9   1   1     0.014       4.3    5.7  10.9    35   118   355   438   310   454 0.53 - Protein of unknown function (DUF1777)
